
JOURNAL INFORMATION
==============================
NLM Title Abbreviation: Behav Neurol
Journal ID: Behav Neurol
NLM Journal ID: 8914585
Journal ID: BN

Behavioural Neurology

ISSN: 0953-4180
EISSN: 1875-8584
Publisher: Wiley

ARTICLE INFORMATION
==============================
PMCID: PMC5294269
PMID: 22713382
DOI: 10.3233/BEN-2012-119000
Article ID: 950972
Article version: 1
Subjects: Introduction

Acquired Dyslexia and Dysgraphia across Scripts

Weekes Brendan Stuart *
Laboratory of Communication Science University of Hong Kong Hong Kong China
*Brendan Stuart Weekes: weekes@hku.hk
Print publication date: 2012
Electronic publication date: 2012 Mar 19
Volume: 25
Issue: 3
First page: 159
Last page: 163
Received 2012 Mar 16; Accepted 2012 Mar 16
Copyright: Copyright © 2012 Hindawi Publishing Corporation and the authors.
Copyright year: 2012
License: This is an open access article distributed under the Creative Commons Attribution License, which permits unrestricted use, distribution, and reproduction in any medium, provided the original work is properly cited.
License URL: https://creativecommons.org/licenses/by/3.0/

Keywords: Models of reading, Spelling, Biscript reading, Bilingualism

==============================
Behavioural Neurology 25 (2012) 159–163
DOI 10.3233/BEN-2012-119000
IOS Press

### Introduction

### Acquired dyslexia and dysgraphia across scripts

Brendan Stuart Weekes
*Laboratory of Communication Science, University of Hong Kong, Hong Kong, China*
*Tel.: +852 2241 5986; Fax: +852 2241 5988; E-mail: weekes@hku.hk*

Keywords: Models of reading, Spelling, Biscript reading, Bilingualism

Many studies have investigated the cognitive processes used to read and to spell in English. Much of our kowledge about these processes comes from reports of patients with acquired dyslexia and dysgraphia, first identified in the behavioral neurology clinic. As in all clinical studies, the detail in these reports includes description of preserved and impaired abilities of the patient, usually with reference to a cognitive neuropsychological model of reading and spelling [6,12,13,26, 28,29]. The beauty of these reports is the insight provided to the clinician and to theorist about the topography of cognitive processing, allowing science to 'carve nature at its joints'.

One criticism of this research is the emphasis on European languages. However, a review of acquired dyslexia and dysgraphia in languages other than English reveals an interesting fact. Many cases are reported in Behavioral Neurology – a revelation that reflects the global reach and sophistication of this readership. It is therefore a great privilege to present this Special Issue on the topic of Reading and Writing Disorders Across Scripts.

The question posed to the authors (and readers) of the papers in this Special Issue is whether the processes identified for reading and spelling in cognitive neuropsychological models developed for English, extend to other languages. This is not a trivial question that is limited to the armchair. Language processing in bilingual speakers is moving towards the front of cognitive neuropsychological enquiry simply because bilingual speakers are the majority of speakers in many language environments. Thus, it is of interest to know about dyslexia and dysgraphia in Chinese, Korean and Urdu for theoretical reasons but also because it is necessary to know if the cognitive processes used to read and to spell in one language have any effects on reading and spelling in another language [see 8, 9, 16 for interesting answers]. If the cognitive mechanisms used to read and to spell in one language impact on reading and spelling in another language then several clinical implications follow, specifically for the treatment of acquired dyslexia and dysgraphia in bilingual speakers [23,26, 40].

The contributors to this Special Issue report on patterns of acquired dyslexia and dysgraphia in Arabic, Chinese, Greek, Hebrew, Hungarian, Italian, Japanese, Russian, Slovak and Spanish. One unique feature is reports of multilingual speakers with reading and spelling disorders in different languages (Druks et al.; Friedmann and Haddad; Kambanaros et al.; Senaha and Parente). These cases not only highlight the striking similarities in the patterns of dyslexia and dysgraphia across languages, they converge on the view that cognitive processes used to read and to spell are independent of linguistic differences. Such reports have implications for theoretical models of bilingual language processing because they suggest language 'non-selective' access to the reading system. For example, the Bilingual Interac-

ISSN 0953-4180/12/$27.50 © 2012 – IOS Press and the authors. All rights reserved tive Activation (BIA) model developed by Dijkstra and colleagues [8,9] assumes an integrated system for oral reading across languages with language non-selective access to the oral reading system [see also 28, 29].

Wilson, Kahlaoui and Weekes [40] reviewed extant reports of acquired disorders of reading and spelling in bilingual speakers. Their review found that the error patterns of surface, phonological and deep dyslexia in monolingual speakers in different languages are observed in bilingual speakers [1,2,11,14,15,19,20,24, 30,39]. That is not to say that all cases of acquired dyslexia in bilingual speakers show the same pattern of oral reading impairment in both languages. Weekes et al. [38] report Mongolian-Chinese bilingual speakers with different reading errors in two types of script. The Mongolian language has an alphabetic script with a set of sublexical mappings from orthography to phonology. Weekes et al. observed semantic oral reading errors in Mongolian but semantically-related translation errors in Chinese i.e. reading a Chinese character using a semantically-related Mongolian syllable. One patient produced within-language semantic oral reading errors in Mongolian e.g. table read as "stool", but did not produce these errors in Chinese. Language selective reading errors are a challenge for the BIA+ model.

Language selective reading errors are observed in other bilingual speakers. Laine et al. [36] reported a Swedish-Finnish speaker who had acquired dyslexia in both alphabetic scripts but was more likely to make phonological errors when reading Swedish than Finnish. García-Caballero et al. [25] reported a Galician-Spanish patient who had reading difficulties only for Galician – a transparent orthography that shares morphology and vocabulary with Portuguese. Raman and Weekes [30] reported a Turkish-English speaker who was surface dyslexic in English but deep dysgraphic in Turkish. Such reports suggest that oral reading problems manifest themselves differently across languages, according to the unique properties of a script.

It is of interest that language selective oral reading errors are not limited to bilingual speakers. The most striking cases are reports of selective reading impairments in biscriptal patients showing that the type of script used within a language can have an impact on acquired dyslexia. Korean uses two different scripts: an ideographic writing system Hanja and syllabic writing system Hangul. Disorders of reading and writing for ideographic and syllabic characters in Korean can be script specific [18]. These reports are difficult to reconcile with non-selective models of bilingual oral reading [8]. Indeed, Caramelli et al. [4] observed a within language dissociation in reading errors across script in a Brazilian Portuguese-Japanese (Nisei) bilingual speaker who produced more oral reading errors in Japanese (ideographic) than Portuguese (alphabetic) even though he acquired both languages at an early age. Of even more interest was the differential pattern of reading impairment within the Japanese language for Kanji and Kana script. Japanese script uses an ideographic writing system (Kanji) adopted from Chinese characters and a syllabic system (Kana). Reports of monolingual Japanese speakers with acquired dyslexia who read well in one script and not the other i.e. biscriptal dyslexia are plentiful [7,32–34]. However, Caramelli's report was the first to show that acquired dyslexia within Japanese transfers to irregularly spelled Portuguese words (see also Senaha and Parente, this volume).

A review of the cases reported with acquired dyslexia and dysgraphia in bilingual speakers is summarized in Table 1 (biscriptal cases are omitted). Each case is described in terms of the language status i.e. the first acquired (L1) and the second acquired (L2) language, the cerebral pathology (if reported), the pattern of reading impairment and the characterisation of acquired dyslexia stated in the case report. The summary shows a mixed pattern. For some cases there are no differences in quantity or quality of oral reading errors across languages, even if the languages use very different scripts (e.g., Arabic-Hebrew). However for the majority of cases there is some effect of language status on performance with different patterns of reading errors across languages. Two features that emerge from the summary are that 1) effects of language status do not necessarily favour the first acquired language and 2) effects of language status do not depend on similarity between scripts. For example, there are reports of Chinese-English speakers who produce more errors in Chinese (L1) than English (L2) but another report shows the opposite pattern. Reports of acquired dyslexia and dysgraphia in bilingual and biscript patients will continue to challenge and inform cognitive neuropsychological models of reading as will cases of reading and writing disorders in monolingual speakers such as those reported here by Crepaldi et al., Hricova et al., Mondini et al., Semenza et al., and Wilson et al.

In closing, I would very much like to thank Argye Hillis and Naama Friedmann for encouragement and help in editing this Special Issue. Thanks also to the contributors for their insights into acquired disorders of reading and writing across scripts.

Table 1

Reported cases of dyslexia in bilingual speakers (1938–2011)

| Citation | Language pairs | Neuropathology | Errors | Characterisation |
|---|---|---|---|---|
| Lyman et al. (1938) | Chinese-English | Left TBA | More reading errors in English (L2) than Chinese (L1) | None |
| Eng (1998) | Chinese-English | Left TBA | More reading errors in Chinese (L1) than English (L2) | Semantic errors in both languages including translation errors |
| Eng and Obler (2002) | Chinese-English | Left TBA | More reading errors in Chinese (L1) than English (L2) | Semantic and surface errors in both languages (stress and tonal) |
| Byng et al. (1984) | Devanagari-English | Left TBA | More reading errors in Devanagari (L1) than Engish (L2) | None |
| Kambanaros et al. (2012) | Greek-English | Left temporal CVA | More writing errors in English (L2) than Greek | Phonological dysgraphia |
| *Chengappa et al. (2004) | Hindi-English | None reported | More reading errors in English (L2) than in Kannada (L1). | Surface dyslexia in English. |
| Druks et al. (2012) | Hungarian-English | Dementia (nfPPA) | More reading errors in English (L2) than Hungarian (L1). | Progressive dyslexia in both languages |
| Ohno et al. (2002) | Japanese-English | Left hemisphere CVA | More reading errors in Japanese than English (including translation of kana words). Writing spared | Pure alexia for Japanese Kana and Kanji. |
| *Karanth (2002) | Kannada-English | None reported | More reading errors in English (L2) than in Kannada (L1). | Surface dysgraphia in both languages |
| Ratnavalli et al. (2000) | Kannada-English | Left occipital lobe | No differences between languages; writing preserved | Pure alexia in both languages (writing intact) |
| Ratnavalli et al. (2000) | Kannada-English | Left parietal lobe | More reading errors in Kannada (L1); writing difficulties for both languages | None |
| Kim, Na and Park (2007) | Korean-English | Left hemisphere CVA | Dysgraphia in both languages transposition errors between consonants and vowels occurred in English | Graphemic buffer impairment. |
| Masterson et al. (1985) | Spanish-English | Left temporal CVA | More reading errors in English (L2) than Spanish (L1) | None |
| Laganaro & Venet (2001) | Spanish-English | Large left TBA | No differences between languages: more difficulty reading nonwords compared to words. | A mixture of pure and phonological alexia. |
| Raman & Weekes (2005) | Turkish-English | Left temporal CVA | More reading errors in English (L2) than Turkish (L1) | Surface dyslexia in English/deep dysgraphia in Turkish |
| Beaton & Davies (2007) | Welsh-English | Left CVA | More reading errors in English (L2) than Welsh (L1) | Deep dyslexia in both languages |
| Tainturier et al. (2012) | Welsh-English | Left CVA (x7) | No difference between languages | Phonological dyslexia in both languages |
| García-Caballero (2007) | Galician-Spanish | Basal ganglia | Reading errors in Galician (L1) only | None |
| Laine et al. (1994) | Finnish-Swedish | Left anterior CVA | No differences between languages: more difficulty reading nonwords compared to words. | A mixture of pure and phonological alexia. |
| Béland & Mimouni (2001) | Arabic-French | Left temporal CVA | More reading errors in French (L2) than Arabic (L1) | Deep dyslexia in both languages |
| Ibrahim (2008) | Arabic-Hebrew | Left hemisphere tumour | More reading errors in Arabic (L1). Could only spell single words in Arabic | Letter-by-letter reading |
| Ibrahim (2009) | Arabic-Hebrew | Herpes encephalitis | More reading errors in Hebrew (L2). In spelling to dictation, Arabic better preserved than Hebrew | Letter-by-letter reading |
| Friedmann & Haddad | Arabic Hebrew | Left TBA | No difference between languages | Letter position dyslexia |
| Weekes et al. (2007) | Mongolian-Chinese | Left temporal CVA | More reading errors in Chinese (L2) than Mongolian (L1). Translation reading errors | Deep dyslexia in Mongolian and Chinese, |
| Meguro et al. (2003) | Portuguese-Japanese | Dementia (DAT) | More reading errors in Kanji than kana (L2) and irregularly spelled words than nonwords in Portuguese (L1) | Surface dyslexia |
| Senaha & Parente (2012) | Portuguese-Japanese | Left traumatic brain injury | More reading errors in Kanji than kana (L2) and irregularly spelled words than nonwords in Portuguese (L1) | Surface dyslexia |
| Caramelli et al. (1994) | Portuguese-Japanese | Left traumatic brain injury | More reading errors in Japanese (L2) than Portuguese (L1). More reading impairment for Kanji than Kana | None |
| Meguro et al. (2003) | Portuguese-Japanese | Dementia (Alzheimer) | Kana, Portuguese and Japanese nonwords > Portuguese irregular words (L1) > Kanji characters (L2) | None |
| *Wydell & Butterworth | English-Japanese | None reported | More reading errors in English (L1) than Japanese (Kanji and Kana) | Developmental phonological dyslexia in English |

*These are cases of developmental dyslexia where no neuropathology is reported.

### References

[1] A. Beaton and W. Davies, Semantic errors in deep dyslexia: Does orthographic depth matter? *Cognitive Neuropsychology* **24**(3) (2007), 312–323.

[2] R. Beland and Z. Mimouni, Deep dyslexia in the two languages of an Arabic/French bilingual patient, *Cognition* **82** (2001), 77–126.

[3] S. Byng, M. Coltheart, J. Masterson, M. Prior and J. Riddoch, Bilingual biscriptal deep dyslexia, *The Quarterly Journal of Experimental Psychology Section A* **36**(3) (1984), 417–433.

[4] P. Caramelli, M. Parente, M.L. Hosogi, M. Bois and A.R. Lecours, Unexpected reading dissociation in a Brazilian Nisei with crossed aphasia, *Behavioral Neurology* **7** (1994), 156–164.

[5] S. Chengappa, S. Bhat and P. Padakannaya, Reading and writing skills in multilingual multiliterate aphasics: Two case studies, *Reading and Writing* **17**(1–2) (2004), 121–135.

[6] M. Coltheart, K. Rastle, C. Perry, R. Langdon and J. Ziegler, DRC: A dual route cascaded model of visual word recognition and reading aloud, *Psychological Review* **108** (1) (2001), 204–256.

[7] F. Cremaschi and E. Dujovny, Japanese language and brain localization, *Neurological Research* **18**(3) (1996), 212–216.

[8] T. Dijkstra and W.J.B van Heuven, The architecture of the bilingual word recognition system: From identification to decision, *Bilingualism: Language and Cognition* **5** (2002), 175–197.

[9] T. Dijkstra, M. Timmermans and H. Schriefers, On being blinded by your other language: Effects of task demands on interlingual homograph recognition, *Journal of Memory and Language* **42** (2000), 445–464.

[10] N. Eng and L.K. Obler, Acquired dyslexia in a biscript reader following traumatic brain injury: A second case, *Topics in Language Disorders* **22**(5) (2002), 5–19.

[11] A. Garcia-Caballero, I. Garcia-Lado, J. Gonzalez-Hermida, R. Areu, M.J. Recimil, O. Juncos Rabadan, S. Lamas and F.J. Jorge, Paradoxical recovery in a bilingual patient with aphasia after right capsuloputaminal infarction, *Journal of Neurology, Neurosurgery and Psychiatry* **78** (2007), 89–91.

[12] A. Hillis and A. Caramazza, Converging evidence for the interaction of semantic and phonological information in accessing lexical information for spoken output, *Cognitive Neuropsychology* **12** (1995), 187–227.

[13] G. Houghton and M Zorzi, Normal and impaired spelling in a connectionist dual-route architecture, *Cognitive Neuropsychology* **20**(2) (2003), 115–162.

[14] R. Ibrahim, Performance in L1 and L2 observed in Arabic-Hebrew bilingual aphasia following brain tumour: a case constitutes double dissociation, *Psychology Research and Behaviour Management* **11** (2008), 11–19.

[15] R. Ibrahim, Selective deficit of second language: a case study of a brain damaged Arabic-Hebrew bilingual patient, *Behavioural and Brain Functions* **5** (2009), 1–10.

[16] D. Jared and J.F. Kroll, Do bilinguals activate phonological representations in one or both of their languages when naming words? *Journal of Memory and Language* **44** (2001), 2–31.

[17] P. Karanth, The search for deep dyslexia in syllabic writing systems, *Journal of Neurolinguistics* **15**(2) (2002), 143–155.

[18] H. Kim, D.L. Na and E.S. Park, Intransigent vowel-consonant position in Korean dysgraphia: Evidence of spatial-con- structive representation, *Behavioural Neurology* **18** (2007), 91–97.

[19] M. Laine, J. Niemi, P. Koivuselkä-Sallinen, E. Ahlsén and J. Hyönä, A neurolinguistic analysis of morphological deficits in a Finnish-Swedish bilingual aphasic, *Clinical Linguistics and Phonetics* **8** (1994), 177–200.

[20] M. Laganaro and M. Overton-Venet, Acquired alexia in multilingual aphasia and computer-assisted treatment in both languages: issues of generalisation and transfer, *Folia Phoniatrica et Logoaedica* **53** (2001), 135–144.

[21] M. Laine, P. Neimi, J. Neimi and P. Koivuselkä-Sallinen, Semantic errors in deep dyslexia, *Brain and Language* **38** (1990), 207–214.

[22] M. Laine, J. Niemi, P. Koivuselkä-Sallinen, E. Ahlsén and J. Hyönä, A neurolinguistic analysis of morphological deficits in a Finnish-Swedish bilingual aphasic, *Clinical Linguistics and Phonetics* **8** (1994), 177–200.

[23] B. Lorenzen and L. Murray, Bilingual aphasia: a theoretical and clinical review, *American Journal of Speech-Language Pathology* **17** (2008), 299–317.

[24] J. Masterson, M. Coltheart and P. Meara, Surface dyslexia in a language without irregularly spelled words, in: *Surface Dyslexia: Neuropsychological and Cognitive Studies of Phonological Reading*, K. Patterson, J.C. Marshall and M. Coltheart, eds, Hove, UK: Lawrence Erlbaum, 1985, pp. 215–223.

[25] M. Meguro, M.L.H. Senaha, P. Caramelli, J. Ishizaki, R.Y.S. Chubacci, H. Ambo, R. Nitrini and A. Yamadori, Language deterioration in four Japanese-Portuguese bilingual patients with Alzheimer's disease: a trans-cultural study of Japanese elderly immigrants in Brazil, *Psychogeriatrics* **3**(2) (2003), 63–68.

[26] L.K. Obler, Dyslexia in bilinguals, in: *Dyslexia: A Global Issue*, R.N. Malatesha and H.A. Whitaker, eds, The Hague: Martineau and Nijhoff, 1983, pp. 477–496.

[27] T.K. Ohno, S. Takeda, S. Kato and S. Hirai, Pure alexia in a Japanese-English bilingual: dissociation between the two languages, *Journal Neurological Science* **249** (2002), 105–107.

[28] D.C. Plaut, J.D. McClelland, M.S. Seidenberg and K. Patterson, Understanding normal and impaired word reading: Computational principles in quasi-regular domains, *Psychological Review* **103** (1996), 56–115.

[29] D.C. Plaut, Graded modality-specific specialization in semantics: A computational account of optic aphasia, *Cognitive Neuropsychology* **19**(7) (2002), 603–639.

[30] I. Raman and B.S. Weekes, Acquired dyslexia and dysgraphia in a biscriptal Turkish-English Reader, *Annals of Dyslexia* **55**(1) (2005), 71–96.

[31] E. Ratnavalli, G. Geetha Murthy, D. Nagarajaa, M. Veerendrakumara, M. Jayaramb and P.N. Jayakumarc, Alexia in Indian bilinguals, *Journal of Neurolinguistics* **13**(1) (2000), 37–46.

[32] Y. Sakurai, T. Momose, M. Iwata, Y. Sudo, K. Ohtomo and I. Kanazawa, Different cortical activity in reading of Kanji words, Kana words and Kana nonwords, *Cognitive Brain Research* **9** (2000), 111–115.

[33] Y. Sakurai, M. Asami and T. Mannen, Alexia and agraphia with lesions of the angular and supramarginal gyri: Evidence for the disruption of sequential processing, *Journal of the Neurological Sciences* **288**(1–2) (2010), 25–33.

[34] M. Satoh, K. Takeda and S. Kuzuhara, Agraphia in intellectually normal Japanese patients with ALS: omission of Kana letters, *Journal of Neurology* **256** (2009), 1455–1460.

[35] M.L.H. Senaha and M.A.M.P. Parente, Acquired dyslexia in three writing systems: study of a Portuguese-Japanese bilingual aphasic patient, *Behavioural Neurology*, (in press).

[36] M.J. Tainturier, J. Roberts, S. Schiemenz and E.C. Leek, Do reading processes differ in transparent vs. opaque orthographies? A study of acquired dyslexia in Welsh/English bilinguals, (in press).

[37] B.S. Weekes, Acquired disorders of reading and writing: Cross-script comparisons, *Behavioural Neurology* **16** (2005), 51–57.

[38] B.S. Weekes, I.F. Su, W.G. Yin and X.H. Zhang, Oral reading in bilingual aphasia: evidence from Mongolian and Chinese, *Bilingualism: Language and Cognition* **10**(2) (2007), 201–210.

[39] B.S. Weekes and I. Raman, Bilingual deep dysphasia, *Cognitive Neuropsychology* **25**(3) (2008), 411–436.

[40] M.A. Wilson, K. Kahlaoui and B.S. Weekes, Acquired dyslexia and dysgraphia in bilinguals across alphabetical and non-alphabetical scripts, in: *Multilingual Aphasia*, M. Gitterman and L. Obler, eds, *Multilingual Matters*, Clevendon, UK, 2012.
